# Comprehensive genomics and expression analysis of eceriferum (CER) genes in sunflower (*Helianthus annuus*)

**DOI:** 10.1016/j.sjbs.2021.07.077

**Published:** 2021-08-02

**Authors:** Hafiz Muhammad Ahmad, Xiukang Wang, Sajid Fiaz, Mahmood-Ur-Rahman, Muhammad Azhar Nadeem, Sher Aslam Khan, Sunny Ahmar, Farrukh Azeem, Tayyaba Shaheen, Freddy Mora-Poblete

**Affiliations:** a Department of Bioinformatics and Biotechnology, Government College University, Faisalabad, Pakistan; b College of Life Sciences, Yan’an University, Yan’an 716000, Shaanxi, China; c Department of Plant Breeding and Genetics, The University of Haripur, 22620, Pakistan; d Faculty of Agricultural Sciences and Technologies, Sivas University of Sciences and Technology, Sivas 58140, Turkey; e Institute of Biological Sciences, Campus Talca, Universidad deTalca, Talca 3465548, Chile

**Keywords:** Sunflower, *CER* genes, Genome wide analysis, Drought stress, Wax biosynthesis, MW, Molecular weight, PI, Isoelectric point, a.a, Amino acids, CER, Eceriferum, At, *Arabidopsis thaliana*, Han, *Helianthus annuus*, VLCFA, Very long chain fatty acids, VLCA, Very long chain alkanes

## Abstract

Sunflower occupies the fourth position among oilseed crops the around the world. Eceriferum (CER) is an important gene family that plays critical role in very-long-chain fatty acids elongation and biosynthesis of epicuticular waxes under both biotic and abiotic stress conditions. The aim of present study was to investigate the effect of sunflower *CER* genes during drought stress condition. Thus, comparative analysis was undertaken for sunflower CER genes with *Arabidopsis* genome to determine phylogenetic relationship, chromosomal mapping, gene structures, gene ontology and conserved motifs. Furthermore, we subjected the sunflower cultivars under drought stress and used qRT-PCR analysis to explore the expression pattern of CER genes during drought conditions. We identified thirty-seven unevenly distributed CER genes in the sunflower genome. The phylogenetic analysis revealed that *CER* genes were grouped into seven clades in *Arabidopsis*, Helianthus annuus, and *Gossypium hirsutum*. Expression analysis showed that genes *CER10* and *CER60* were upregulated in sunflower during drought conditions, indicating that these genes are activated during drought stress. The results obtained will serve to characterize the *CER* gene family in sunflower and exploit the role of these genes in wax biosynthesis under limited water conditions*.*

**Key message:**

Cuticular waxes protect the plants from drought stress, so we observed the expression of wax bio synthesis genes in recently sequences genome of *Helianthus annuus*. We observed that expression of wax biosynthesis genes *CER10* and *CER60* was upregulated when the plants were subjected to drought stress.

## 1 Introduction

The primary origin of the sunflower (Helianthus annuus) is North America, from where it spread throughout the world (Blackman et al., 2011, Schilling and Heiser, 1981). *H. annuus* exhibits variation in its genome size hence, difference is exist at ploidy levels, it contains diploid (2n = 2x = 34), tetraploid (2n = 4x = 68) and hexaploid species (2n = 6x = 102) with basic chromosome number 17 (Rieseberg and Seiler, 1990). *H. annuus* is an important oilseed crop however some species are used for ornamental purpose only. This crop is also a source of animal feed, and its husk is used in paper industry. *H. annuus* genome was completely sequenced in 2017 (https://sunflowergenome.org/) having estimated genome size of 3.6 gigabases (Badouin et al., 2017). Epicuticular waxes are made up from mixture of very long chain lipids (VLCL) which are derived from fatty acids as results of Acyl-CoA elongation activities. Cuticular wax seals the areal parts of land plants to protect them from environmental stresses and maintain the water balance by controlling the non-stomatal water loss. Cuticular waxes protect plants from insects, pathogens, bacterias and ultraviolet radiations (Ahmad et al., 2020, Liu et al., 2021) demesnes the dust retention, deposition of water on plant surface, control air pollutants and pollens (Kerstiens, 1996). Plant leaves possessing low wax contents have been reported high transpiration rate and excessive water loss compared to waxy leaves (Muhammad Ahmad et al., 2021).

The term eceriferum (CER) is derived from Latin word “eceriferum” meaning without wax and was coined by [81] who reported the wax mutants in *A. thaliana*. Previous studies has proved that CER1 protein convert the aldehyde to alkanes and is a key component of very-long-chain-alkane (VLCA) synthesis. This protein actively participates in wax biosynthesis and enhancement of pollen fertility. *CER1* gene is activated in response to biotic and abiotic stress (Aarts et al., 1995, Bourdenx et al., 2011, Bernard and Joubès, 2013). *CER2* gene is localized in endoplasmic reticulum where it performs regulatory functions and participate in very-long-chain-fatty-acid (VLCFA) elongation process. It also functions as acyltransferase in C28 elongation mechanism (Haslam et al., 2015, Jenks et al., 1995, Wang et al., 2017). Other functions of CER2 protein are formation of pollen coat and cuticles (Haslam et al., 2015). Major roll of CER3 protein is formation of cuticle membrane and biosynthesis of cuticular wax. This protein also functions as fatty acid reductase and is responsible for alkane production and aldehyde formation. CER3 interact with CER1 and catalyze the redox dependent VLCA from very-long-chain-Acyl-CoA’s (VLC Acyl-CoA’s) (Bernard and Joubès, 2013, Chen et al., 2003). *CER4* genes are expressed in plant leaves, stems, siliques, flowers, and roots. Major function of these genes is fatty acid biosynthesis and cuticular wax formation (Qu et al., 2017). These genes encoded an alcohol-forming fatty Acyl-CoA reductase. Products of *CER4 and CER6* genes actively participate in fatty aldehyde reduction and C26 fatty Acyl-CoA elongation, respectively (Doan et al., 2009). Epicuticular wax is formed in epidermal cells and transporters are required for cutin and wax secretion from epidermal cells to cuticle (Rowland et al., 2006, Panikashvili et al., 2007). *CER5* genes affects cutin metabolism in reproductive organs and suberin in roots (Panikashvili et al., 2010) along with the export of diverse cuticular lipids and secretion of wax (McFarlane et al., 2014). *CER5* genes also resist downy mildew infection and regulates callose deposition in infectious plants (Caillaud et al., 2014). *CER6* gene is required for fatty acid elongation from C26 for wax biosynthesis in epidermis and root hair development (Pang et al., 2010). This gene also plays essential roll suberin biosynthesis and pollen fertility under Acyl-reduction and de-carbonylation wax biosynthesis pathways (Fiebig et al., 2000, Millar et al., 1999). CER6 is an important enzyme for condensation of stem wax and lipid biosynthesis for pollen coats (Fiebig et al., 2000). *CER7* involved in the regulation of cuticular wax biosynthesis by controlling the expression of *CER3. CER7* also regulates the biosynthesis of cuticular wax in developing inflorescence stem (Lam et al., 2012). *CER8* plays role in fatty acid metabolism pathways and lead to VLCFA formation by synthesizing cellular lipids. It performs a specific activity against VLCFA with more than 24 carbons (Lü et al., 2009, Weng et al., 2010). Eceriferum9 engaged in maintaining the leaf water status and cuticular wax formation in *A. thaliana* (Lü et al., 2012). These genes is predicted to involve in trihcome papillae development (Suo et al., 2013) transformation of epicuticular wax substrates, synthesis of VLCAF and cell expansion during plant morphogenesis (Zheng et al., 2005). *CER10* is involved in the production of various chain length VLVFAs, which are engaged in various biological activities as a precursors of membrane lipid and lipid mediators (Zheng et al., 2005). Further these genes lay a vital role in lipid storage, sphingolipids and epicuticular waxes biosynthesis (Zheng et al., 2005, Gable et al., 2004). A member of eceriferum family *CER13* is required for release of C30 fatty acid from elongation complex and reduce the fatty acid to aldehyde of similar length. It has been reported that activation of *CER13* expressed ester alcohol pattern with increase in C30 level (Lai et al., 2007). *CER17* is a Acyl desaturase gene and produces cutin monomers and unsaturated primary alcohols (Yang et al., 2017). The function of *CER19* is fatty Acyl-CoA elongation from C28 to C30. While *CER20* is predicted to activate for oxidation of C29 alcohol from c29 alkane (Rashotte et al., 2001). The gene *CER22* is an allelic to *CER1* and in activated under stress conditions for synthesis of wax alkanes (Sakuradani et al., 2013)*.* This gene is localized in plant leaves and is required for elongation of C30 fatty acids to form *VLCFAs* (Sakuradani et al., 2013). Expression of CER26 mutant facilitates the elongation of VLCFAs from C30 to more and involved in EW biosynthesis (Pascal et al., 2019, Pascal et al., 2013). Silencing of *CER26* leads to biosynthesis of a wax monomer namely phenyl propanoid (Hoffmann et al., 2004). Role of eceriferum2-lik protein has been reported in *A. thaliana* and *Zea mays* for cuticular wax biosynthesis. This gene also have role in chain length modification (Haslam et al., 2015). Latest investigated protein of this family is *CER60* which is also involved in fatty acid biosynthesis pathway and synthesis VLCFAs having carbon chain length from 26 to 30 (Trenkamp et al., 2004).

The objective of the present research was to characterize the *CER* genes family across the *H. annuus* and *A. thaliana* genomes. A comprehensive genomic comparison was performed among these species to discover functional similarities by using bioinformatics tools. Further, expression analysis of *CER* genes under drought stress will provide the evidence about the upregulation of wax biosynthesis genes during stress conditions. This research will also reveal functional similarities at genomic and proteomic levels in *A. thaliana* and *H. annuus* about eceriferum gene family.

## 2 Materials and methods

### 2.1 Retrieval of protein sequences and physio-chemical properties of CER genes

Sequences of Eceriferum (CER) family proteins in *A. thaliana* were retrieved from National Center for Biotechnology Information (NCBI) (https://www.ncbi.nlm.nih.gov/) and TAIR (https://www.arabidopsis.org/). Blast-p tool of NCBI was used to find the similar proteins in *H. annuus*. Plant Genome and System Biology (PGSB) (http://pgsb.helmholtz-muenchen.de/plant/plantsdb.jsp) and Phytozome v 11.0 (https://phytozome.jgi.doe.gov/pz/portal.html) data bases were used for further verification of retrieved sequences. Physiochemical parameters of CER proteins such as, amino acid length (a.a), molecular weight (M.W), and theoretical (Pi), were calculated using expasy an online web tool (http://web.expasy.org/cbi-bin/protparam/protpara) (Gasteiger et al., 2005).

### 2.2 Sequence alignment and construction of phylogenetic tree

*A. thaliana, H. annuus* and *G. hersutum* CER proteins were retrieved from NCBI (https://www.ncbi.nlm.nih.gov/) and then aligned by using ClustalX tool (Thompson et al., 1997). We used neighbor joining method to construct the phylogenetic tree (Saitou and Nei, 1987) using MEGA 7 program (Kumar et al., 2016) at 1000 boost strap value.

### 2.3 Conserved domain and gene structure analysis

We used MEME tool of 4.9.1 version (http://meme.nbcr.net/meme/cgi-bin/meme.cgi) to conduct the motif analysis of *H. annuus* and *A. thaliana* CER proteins. Maximum number of motifs were fixed 20, having motif width 5 to 90 residues. However repeatedly occurrence of a single motif among sequences were settled to any number of repetitions.

Gene structure display server (GSD 2) (http://gsds.cbi.pku.edu.cn/) was used to determine the gene structure in *A. thaliana* and *H. annuus* genome by using genomic DNA and CDS sequences as input files. CDSs were represented by yellow lines, introns by thin black lines, upstream/down streams by blue lines and each gene were illustrated in phylogenetic tree at their corresponding place.

### 2.4 Chromosomal mapping of CER genes and synteny analysis

The Arabidopsis information resource (TAIR) (https://www.arabidopsis.org/) was used to determine the exact location of *CER* genes on *A. thaliana* chromosomes. An online tool “Map gene 2chromosome v2” (http://mg2c.iask.in/mg2c v2.0/) was used to investigate the location of *CER* genes in *H. annuus* chromosomes by using gene ID, start and finish location of gene, and corresponding chromosomal sequence length as input files without altering the default settings of the tool.

To determine the evolutionary origin of CER proteins in *H. annuus* and *A. thaliana*, protein sequences retrieved from NCBI were submitted to online synteny tool (circoletto tools.bat.infspire.org/circoletto). The bands were represented with different colors.

### 2.5 Gene ontology

Blast2GO program (Gotz et al., 2008) was used to determine the gene ontology for *H. annuus* and *A. thaliana;* CER proteins. Amino acid sequences were used as input file and default parameters were not changed. Different databases like Swiss-Prot protein, NCBI non-redundant protein (nr), Gene ontology (GO), Kyoto Encyclopedia of Genes (KEGG) protein family and Cluster of Orthologs Groups (COGs) were used for characterization of *CER* genes in both plant species.

### 2.6 Plants material and drought treatment

Three sunflower genotypes, FH-331, FH-629, and FH-630, were cultivated in pods in a growth chamber containing red sandy soil and manure (2:1) to study the expression pattern of *HanCER10* and *HanCER60* genes in *H. annuus* under drought condition. Temperature of growth chamber was maintained (25/22 °C), photoperiod (16-h), and relative humidity of 75%. One month old sunflower plants were subjected to dehydration stress for ten days and then rewatered. Five leaves from each genotype were collected from different locations and were instantly freeze in liquid nitrogen at −80 °C for further analysis.

### 2.7 RNA isolation and RT-qPCR analysis

Total RNA was extracted from frozen samples using TriZol reagents, as directed by the manufacturer. Nanodrop, ND-1000 (Nano Drop Technologies, Inc) was used to measure the concentration of RNA samples. Ambion's DNA free TM-Kit was used to remove DNA contamination from RNA. Primer3 online tool (http://frodo.wi.mit.edu/) was used to design the primers based on prior investigations about *CER* gene involved in epicuticular wax biosynthesis under drought stress (Table 1). Reverse Transcriptase Polymerase Chain Reaction (RT-PCR) was used for amplification and first strand cDNA synthesis. Paired Student's *t*-test was used to evaluate the significance of the differences between the samples, with p-values of *p < 0.05 and **p < 0.01 being considered significant.

**Table 1:** List of forward and reverse primers used for qRT-PCR.

Forward primer for CER10	5′-CTGGGGGCACAAGTTT-3′
Reverse primer for CER10	5′-TGGCAAACCAAACCAA-3′
Forward primer for CER60	5′-GCCATCGAGCTTCTCC-3′
Reverse primer for CER60	5′-TTGGGCCTCGTTTCTT-3′

## 3 Results

In *A. thaliana* 27 *CER* genes and their homologs were searched through computational tools. Detail characterization of *A. thaliana* CER proteins is provided in Table 2. Physiochemical properties of *A. thaliana* CER proteins indicated that these genes were located on all the *A. thaliana* chromosomes. Number of exons ranged from 2 to 19, genes *AtCER6, AtCER27* and *AtCER60* and their homologs bearing 2 exons. Number of amino acids is important tool to describe the stability of a protein. A gene *CER7-2* showed shortest amino acid chain. Isoelectric point is used to determine the net electric charge on proteins. Proteins having isoelectric point below seven are considered as acidic and higher that seven basics. Isoelectric point of *A. thaliana* CER proteins was ranged between 5.41 and 9.49. Ten of the twenty-seven proteins had a PI value below 7, suggesting that they are acidic, whereas the remaining seventeen showed PI value greater than 7, suggesting that these proteins are predominantly basic in nature. CER5-1, CER10-1, CER17-1, and CER60-1 was the highest basic protein having PI more than 9.

**Table 2:** Physiochemical properties of *A. thaliana CER* genes.

**Sr#**	**Gene symbol**	**Gene ID**	**Locus tag**	**Ch #**	**Exon**	**a.a**	**Protein M.W (DK)**	**PI**
1	AtCER1-1	837,602	AT1G02205	1	10	625	72405.83	8.22
2	AtCER1-2	837,602	AT1G02205	1	10	626	72750.2	7.42
3	AtCER1-3	837,602	AT1G02205	1	10	630	73023.58	8.38
4	AtCER1-4	837,602	AT1G02205	1	10	386	44430.2	7.28
5	AtCER1-5	837,602	AT1G02205	1	10	461	52977.51	7.67
6	AtCER22-1	828,553	AT4G24510	4	2	421	47,238	5.38
7	AtCER3-1	835,889	AT5G57800	5	11	632	72288.9	8.78
8	AtCER4-1	829,521	AT4G33790	4	10	493	56034.6	8.78
9	AtCER4-2	829,521	AT4G33790	4	10	380	43367.79	8.9
10	AtCER5-1	841,575	AT1G51500	1	8	687	76450.7	9.33
11	AtCER6-1	843,182	AT1G68530	1	2	497	56395.9	9.08
12	AtCER6-2	843,182	AT1G68530	1	2	377	42723.01	9.02
13	AtCER7-1	820,485	AT3G12990	3	8	307	33874.75	6.16
14	AtCER7-2	820,485	AT3G12990	3	8	221	24625.2	5.88
15	AtCER7-3	820,485	AT3G12990	3	8	287	31709.23	5.69
16	AtCER7-4	820,485	AT3G12990	3	8	307	33874.75	6.16
17	AtCER8-1	819,337	AT2G47240	2	19	660	74597.9	5.97
18	AtCER8-2	819,337	AT2G47240	2	19	601	68143.62	6.04
19	AtCER9-1	829,556	AT4G34100	4	9	1108	123,004	5.97
20	AtCER9-2	829,556	AT4G34100	4	9	1107	122861.7	5.92
21	AtCER10-1	824,702	AT3G55360	3	4	310	35723.6	9.49
22	AtCER17-1	837,146	AT1G06350	1	5	300	35431.8	9.72
22	AtCER22-1	837,602	AT1g02200	1	10	626	72750.2	7.42
23	AtCER22-2	837,602	AT1g02200	1	10	630	73023.58	8.38
24	AtCER22-3	837,602	AT1g02200	1	10	386	44430.2	7.28
25	AtCER22-4	837,602	AT1g02200	1	10	461	52977.51	7.67
26	AtCER27-1	827,018	AT4G13840	4	2	428	47455.4	5.41
27	AtCER60-1	839,131	AT1G25450	1	2	492	55652.9	9.01

By blasting Arabidopsis CER proteins, we find 37 CER genes and their homologs in *H. annuus* genome. Physiochemical properties of these *H. annuus* CER protein presented in Table 3. Results showed that these genes were distributed among all seventeen *H. annuus* chromosomes except 4, 6 and 7. In *H. annuus* number of exons ranged from 1 to 20. Amino acid length ranged from 107 to 1878. Isoelectric point of CER proteins varied from 4.98 to 9.78 indicating that these proteins are acidic as well as basic in nature.

**Table 3:** Physiochemical properties of *CER* genes identified in *H. annuus*.

**Sr#**	**Gene symbol**	**Gene ID**	**Locus tag**	**Ch #**	**Exon**	**a.a**	**Protein M.W (DK)**	**PI**
1	HanCER1-1	110,920,509	HannXRQ_Chr16g0521441	16	10	622	71859.3	7.78
2	HanCER1-2	110,897,561	LOC110897561	13	10	617	71660.41	9.18
3	HanCER1-3	110,912,685	LOC110912685	15	10	622	72557.67	9.07
4	HanCER1-4	110,897,451	LOC110897451	13	10	612	70946.37	8.98
5	HanCER2-1	110,890,844	LOC110890844	11	2	430	47731.8	5.88
6	HanCER2-2	110,908,568	LOC110908568	14	2	429	47887.99	5.87
7	HanCER2-3	110,877,369	HannXRQ_Chr01g0022181	1	2	439	49292.43	5.78
8	HanCER3-1	110,940,683	LOC110940683	5	11	630	71502.4	8.89
9	HanCER3-2	110,910,537	LOC110910537	2	12	650	74693.46	9.1
10	HanCER3-3	110,940,683	LOC110940683	5	11	630	71502.4	8.89
11	HanCER3-4	110,884,239	LOC110884239	10	11	630	72091.02	9.07
12	HanCER4-1	110,904,168	HannXRQ_Chr14g0451501	14	10	492	55554.3	8.07
13	HanCER4-2	110,904,194	LOC110904194	14	10	490	55016.8	8.53
14	HanCER5-1	110,898,430	LOC110898430	13	9	691	76444.1	9.12
15	HanCER5-2	110,878,339	LOC110878339	9	19	676	76096.2	8.68
16	HanCER5-3	110,885,555	LOC110885555	10	40	1850	205,838	5.97
17	HanCER5-4	110,885,555	LOC110885555	10	40	1878	208,886	5.83
18	HanCER6-1	110,911,828	LOC110911828	15	2	496	55769.3	9.21
19	HanCER6-2	110,915,106	LOC110915106	16	1	479	53,941	9.1
20	HanCER6-3	110,886,259	LOC110886259	10	3	550	61,767	9.26
21	HanCER7-1	110,898,428	HannXRQ_Chr13g0401261	13	10	428	46762.5	6.85
22	HanCER7-2	110,915,756	LOC110915756	16	8	367	39965.02	6.52
23	HanCER8-1	110,936,374	HannXRQ_Chr04g0106251	4	19	107	11950.28	8.09
24	HanCER8-2	110,910,053	LOC110910053	15	20	661	74762.22	7.23
25	HanCER8-3	110,930,246	LOC110930246	3	18	661	73503.66	6.07
26	HanCER9-1	110,877,983	LOC110877983	9	8	1051	117646.1	5.09
27	HanCER9-2	110,929,223	LOC110929223	3	9	1081	120883.4	5.76
28	HanCER9-3	110,918,818	LOC110918818	16	8	1024	114463.3	5.87
29	HanCER10-1	110,913,412	LOC110913412	2	4	310	36161.06	9.64
30	HanCER10-2	110,904,732	LOC110904732	14	4	310	36068.9	9.73
31	HanCER10-3	110872584,	LOC110872584	8	4	310	36453.44	9.62
32	HanCER22-2	110,920,509	LOC110920509	16	10	458	52512.88	8.87
33	HanCER22-4	110,912,685	LOC110912685	15	10	548	63913.47	9.3
34	HanCER26-1	110,915,265	LOC110915265	16	2	132	14505.37	4.98
35	HanCER60-1	110,895,450	LOC110895450	12	3	495	55630.07	9.22
36	HanCER60-2	110,923,358	LOC110923358	17	1	471	53078.77	9.09
37	HanCER60-3	110,875,898	LOC110875898	9	2	511	56999.09	9.1

**Fig. 1:**
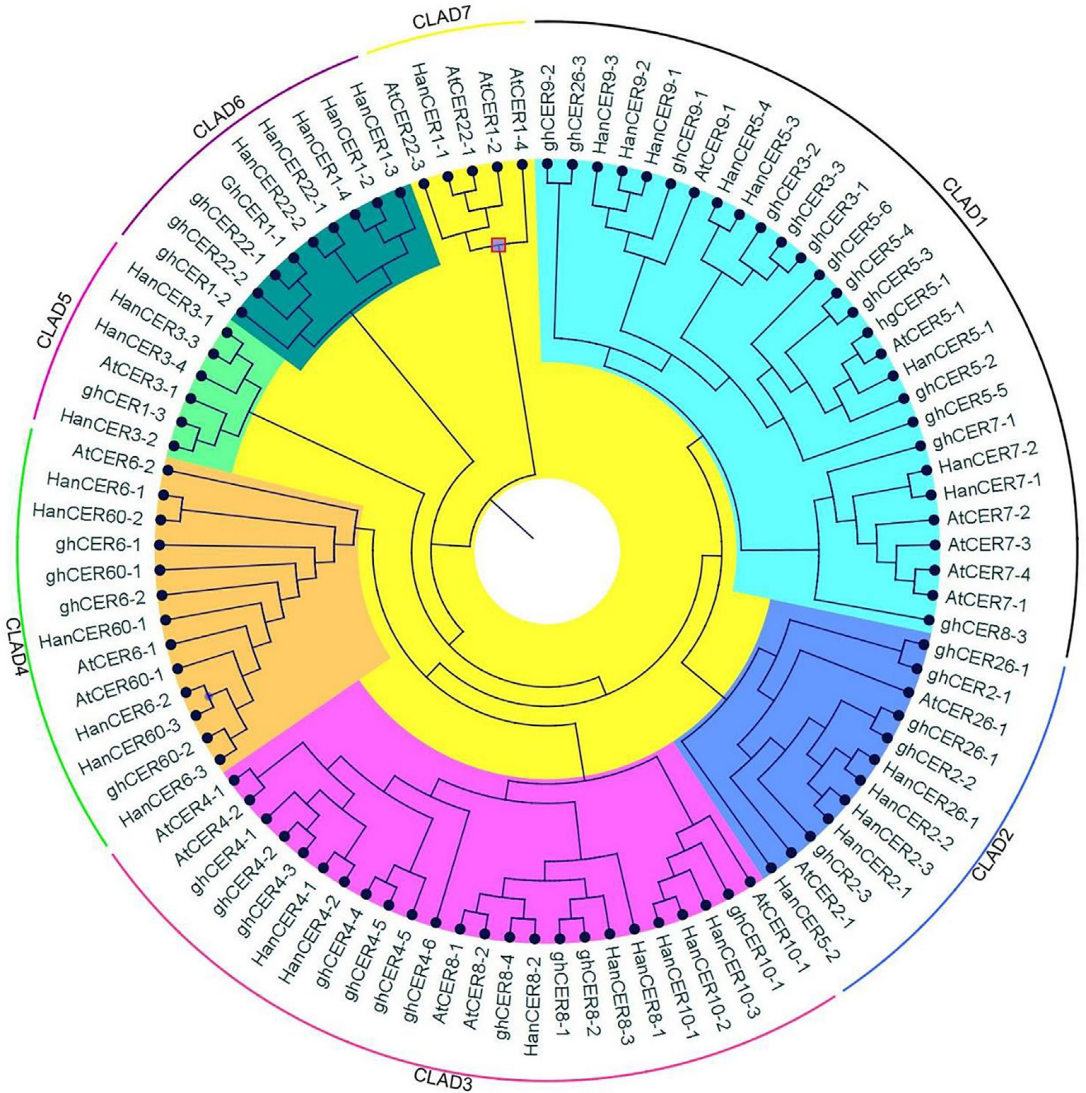
An unrooted phylogenetic tree was constructed by using neighbor joining method on the bases of sunflower, Arabidopsis and cotton CER amino acid sequences with 1000 bootstraps. Sequences were aligned with Clustal X and tree was constructed from aligned sequences by using MEGA 7 tool.

To investigate the evolutionary ancestry and similarity of *CER* family genes in *A. thaliana*, *H. annuus* and *G. hersutum* we constructed an unrooted phylogenetic tree according to neighbor joining method from 102 protein sequences. CER proteins of *A. thaliana*, *H. annuus* and *G. hersutum* were aligned by using ClustalX and phylogenetic tree was constructed by using MEGA7 programme. All position containing missing data and gapes were eliminated. Ninety-four amino acid sequences were used in data to construct the phylogenetic tree. On the bases of phylogenetic tree CER proteins were grouped in seven clads. Genes in same cluster were showing homology between CER protein sequences as shown in Fig. 1. Clad1 (28 genes), clad2 (12 genes), clad3 (24 genes), clad4 (13 genes), clad5 (6 genes), clad6 (9 genes) and clad7 (5 genes). The results indicated that *G. hersutum* CER proteins share great homology with *A. thaliana* and *G. hersutum*. It was noted that in clad 1st to 5th all three species shared the genes. However, in 6th clad no Arabidopsis gene was present and in 7th clad no *G. hersutum* gene contributed that may be due to some special distribution event occurred during the evolutionary process. Similar results of phylogenetic tree were reported in *Cicer arietinum* and *G. hersutum* (Muhammad et al., 2021, Azeem et al., 2018, Waqas et al., 2019). The evolutionary relationship indicate that genes falling in same clad of phylogenetic tree also showed same evolutionary origin which supported the idea of same similar genetic background (Fig. 5). In a recent research (Qi et al., 2019) have reported the similar pattern of *CER* genes in apple plants. Syntenic analysis also confirmed that most the genes falling in same subgroup have same evolutionary origin. Those who doesn’t showed synteny must have passed through complex evolutionary process.

**Fig. 2:**
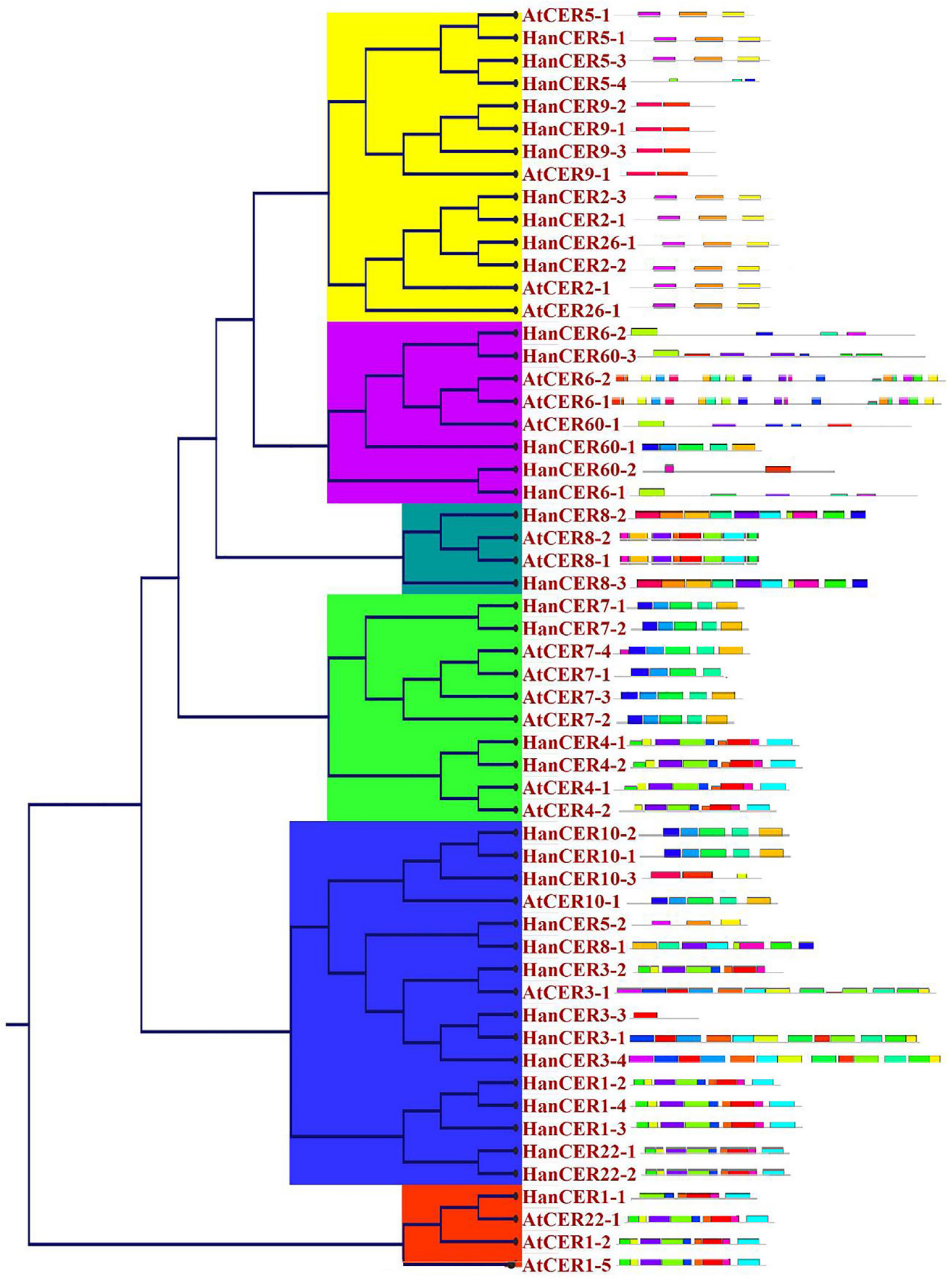
Protein motifs of CER gene proteins were analyzed by online tool MEME (http://meme.nbcr.net/meme/cgi-bin/meme.cgi) which is publically available. The results showed twenty conserved motifs in CER proteins in both plant species. The regular expression of highly conversed motif (Motif 1).

Conserved motif analysis of CER proteins was performed by using online software MEME SUIT (http://meme.nbcr.net/meme/cgi-bin/meme.cgi). The default parameters used for motif discovery were “Maximum number of motifs (10), unlimited motif E-value threshold, minimum and maximum motif width was 6 and 50 respectively. Minimum and maximum sites per motifs were fixed 2 and 175 respectively”. We identified twenty distinct conserved motifs and placed according to the position of genes in phylogenetic tree. The results indicate that pattern of motifs was almost conserved within a clad of phylogenetic tree. Frequently close members in a clad shared common motif composition. Similar results about the conserved motifs have been reported in *C. arietinum* (Waqas et al., 2019) *Vitis vinifera*; *O. sativa* and *A. thaliana* (Wang et al., 2015). Maximum 18 conserved motifs were observed in HanCER3-1 and HanCER3-3 followed by AtCER3-1 and HanCER3-2 proteins. A protein HanCER26-4 was the only protein which did not showed any conserved region. Moreover, the 6th motif was the most commonly occurring motif shown in Fig. 2. Conservation of motifs pattern within subgroup has been reported in *H. annuus* and *Z. mays* genome (Ahmad et al., 2020, Bari et al., 2018).

**Fig. 3:**
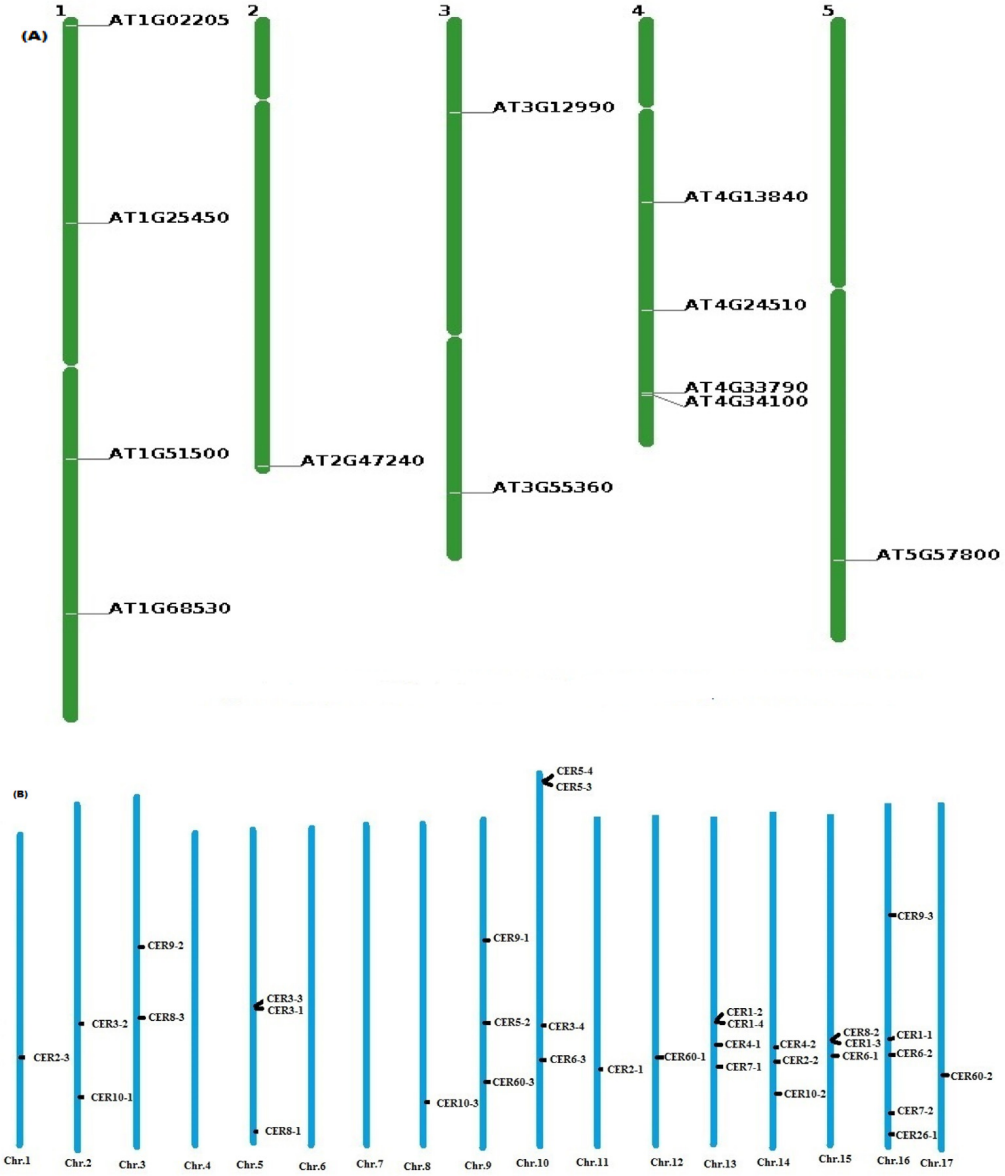
Chromosomal location of *CER* genes in Arabidopsis (a) and Sunflower (b) plants. Green mapping indicating the location of *CER* genes on Arabidopsis chromosomes and blue mapping indicate the location of *CER* genes on sunflower chromosomes.

To find the location of *CER* genes on *A. thaliana* chromosomes we used an online database The Arabidopsis Information Resource “TAIR”. In *A. thaliana CER* genes were located on all 5 chromosomes. Whereas chromosome no. 1 and 4 each possessed 4 genes. One gene was located on chromosome 2 and 5. According to this mapping, two genes were located on the third chromosome. Fig. 3 depicts the distribution of *CER* genes across the several chromosomes of *A. thaliana*. Chromosomal mapping of *H. annuus* was performed by using “Map gene 2chromosome v2”. In *H. annuus* chromosomes maximum five *CER* genes were present on chromosome Number 16 followed by chromosome no. 10 and 13 both bearing four *CER* genes. However, no *CER* gene was reported on chromosome no. 4, 6 and 7 which sported our results presented in Table 2.

**Fig. 4:**
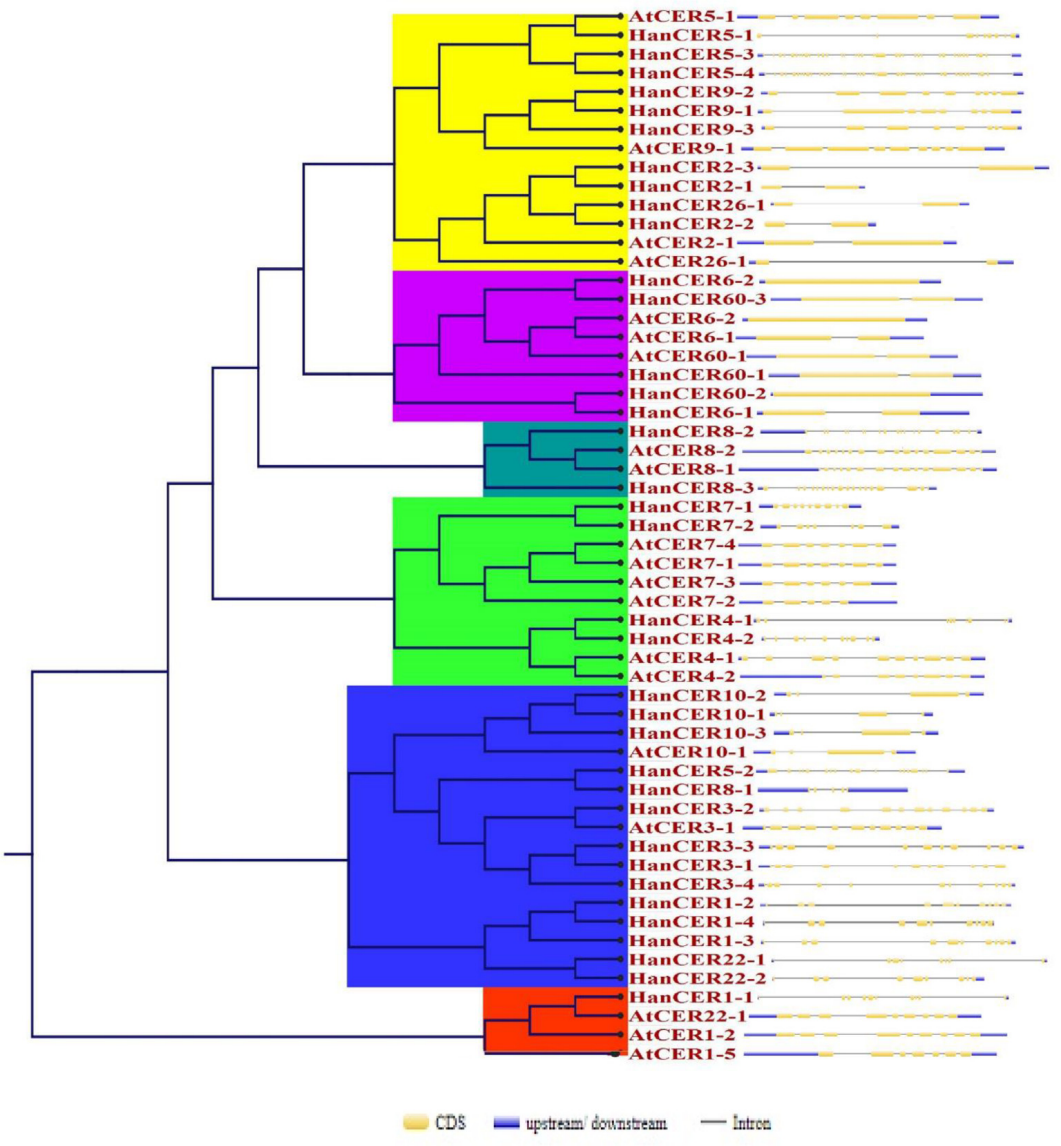
Gene structure analysis of sunflower and Arabidopsis *CER* genes, where.

Intron/exon map helps to understand the structural diversity of multigene families. An online available server “GSDS 2.0) was used to find the intron/exon position in *H. annuus* and *A. thaliana* genome. Detail structural organization of intron/exon presented in Fig. 4. Intron/exons structure of each gene was elaborated at their concerned position in phylogenetic tree. Previously it was observed that multi-exon gene structure allow alternative splicing, which produces messenger RNA and protein isoforms with differing roles (Chen and Manley, 2009). Results of Fig. 4 showed that number of introns/exons varied from 1 to 40 in *A. thaliana* and *H. annuus CER* genes. Two genes *AtCER6-2* and *HanCER6-2* showed no intron. It was also noted that loss and gain in exon numbers occurred during evolution of *CER* gene family which indicated the functional diversity among the closely linked genes. Similar results for WRKY III genes was reported by (Wang et al., 2015).

**Fig. 5:**
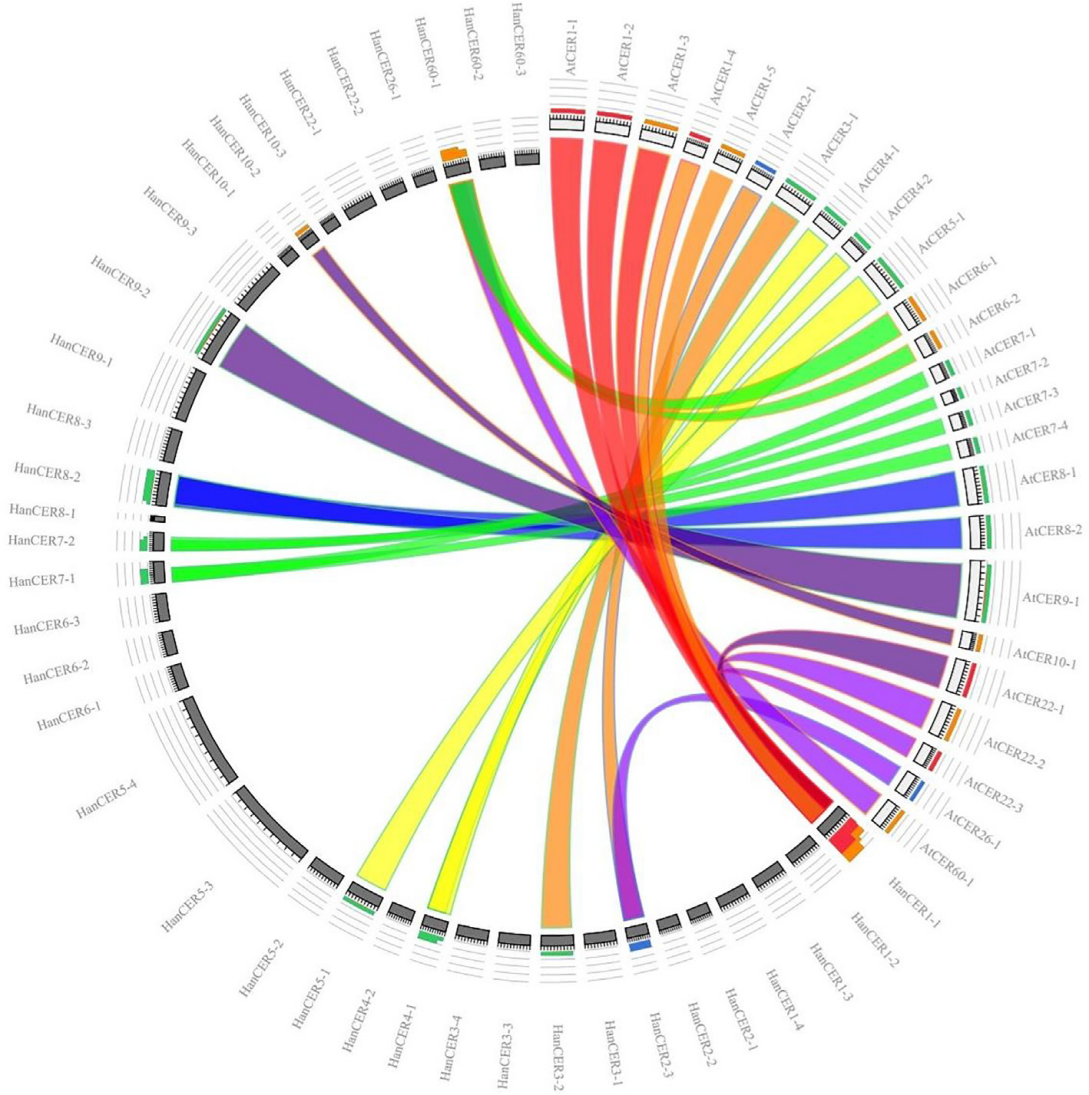
Evolutionary relationship among sunflower and Arabidopsis CER genes. Genes possessing similar color bands dissecting circle at various points indicated that have same evolutionary origin.

A comparative synteny analysis of *H. annuus* and *A. thaliana* CER protein sequences was conducted to get an idea of the origin and evolutionary relationship of the CER protein family genes in both plant species. A synteny analysis was performed between 37 *H. annuus* and 27 *A. thaliana* CER proteins. The proteins from both species were closely associated and exhibited higher resemblance in evolutionary correlation analyses. Although there were specific genes that showed greater similarity than others, as demonstrated in Fig. 5. It was noted that *AtCER1-1* have some evolutionary origin to *HanCER1-1* and its variants. *AtCER9-1* have same evolutionary origin as *HanCER9-2*. Similarly, *AtCER8-1, AtCER8-2* genes showed evolutionary association with *HanCER8-2* and *HanCER8-3. AtCER5-1* and *AtCER4-1* possessed evolutionary similar origin with *HanCER5-1, HanCER4-1* and *HanCER4.*

**Fig. 6:**
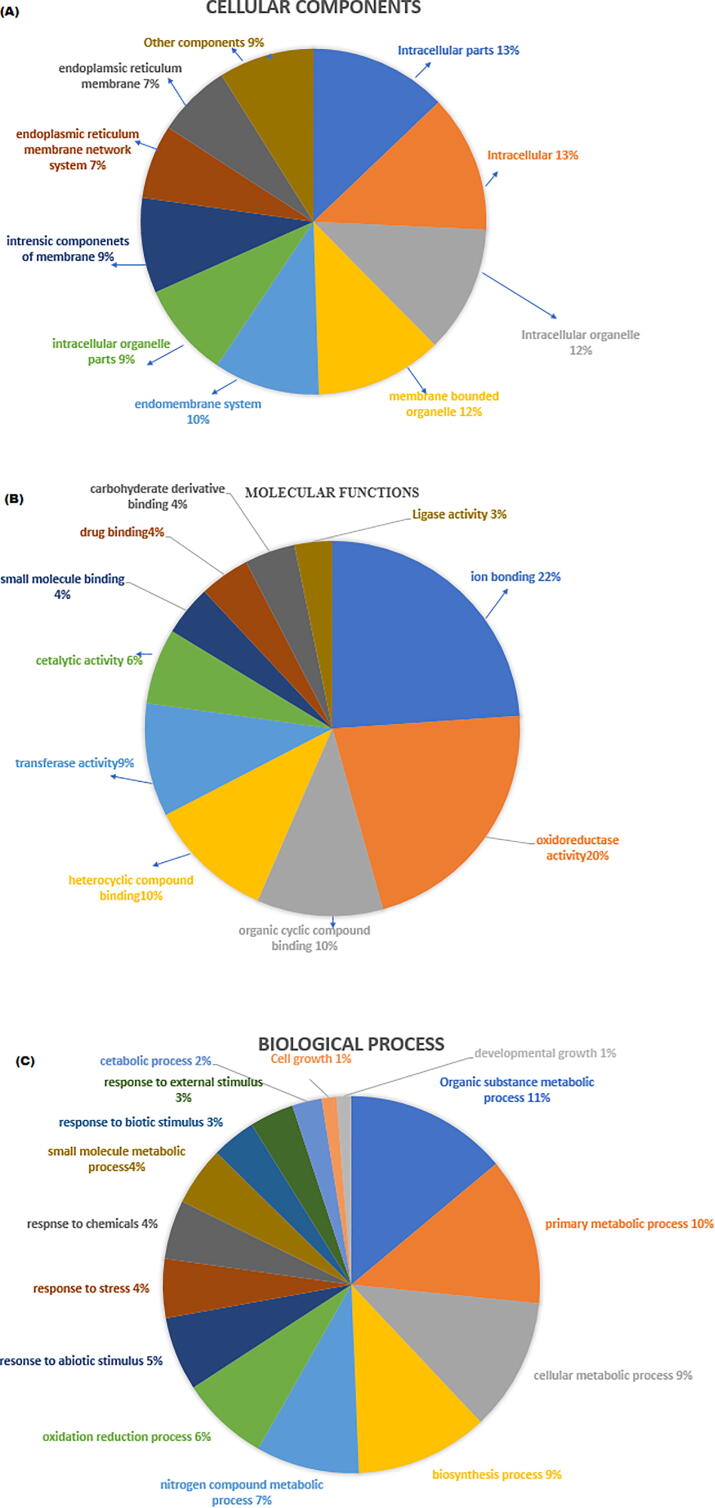
Gene ontology of Cellular components (A), Molecular functions (B) and Biology process (C), based on sixty-four Arabidopsis and sunflower *CER* genes. Each function/process is elaborated with different color and percentage was shown to every corresponding function/process.

Gene ontology (GO) of CER family categorized on the bases of cellular components showed that 13% genes belong to intracellular parts, 12% intracellular organelle and membrane bounded organelle, 10% endomembrane system, 9% intracellular organelle parts, intrinsic components of membrane, 7% nuclear outer membrane-endoplasmic reticulum membrane network, endoplasmic reticulum, 3% catalytic complex, 2% organelle lumen, plasma membrane and cell periphery. Binding or catalysis activities of a genes can be expressed by determining GO of molecular functions. Gene ontology of molecular functions of *CER* genes indicated that 22% of these genes were involved in ionic binding, 20% oxidoreductase activities, 10% organic cyclic compounds binding, 7% heterocyclic compound binding, 9% transferase activities, 6% catalytic activities, 4% small molecule binding, drug binding, carbohydrate derivative binding and 3% ligase activities. Graphical representation of gene ontology is indicated in Fig. 6.

**Fig. 7:**
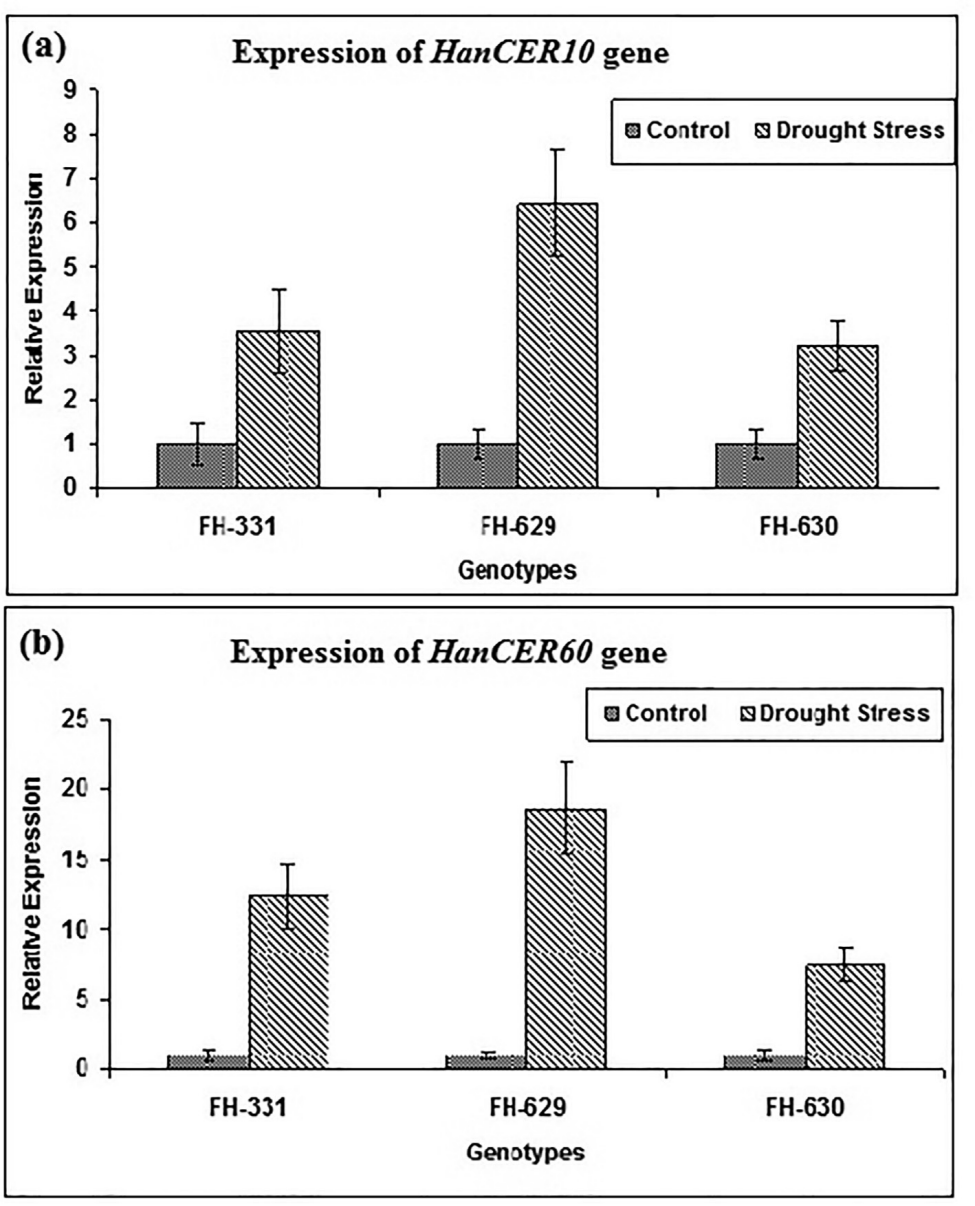
Effects of drought stress on the expression of *CER1, CER60* in sunflower. qRT-PCR was used to examine the expression level of these genes. Student’s *t*-test (P < 0.05) was used to compare the means of three biological and technical replicates. Regularly watered plants were named as control.

To investigate the expression of pattern *HanCER10, HanCER60,* in *H. annuus* leaves qRT-PCR was performed. *CER10, CER60* transcripts were detected in all the *H. annuus* cultivars with diverse expression levels. Results of Fig. 7 showed that transcription level of *CER10, CER60* was higher in drought subjected genotypes as compared to controls which indicated that normally watered plants have less wax load as compared to drought subjected plants. Highest expression of *CER10* and *CER60* was noted in cultivar FH-629 followed by FH-331 and FH630 respectively. Relative expression indicated that expression of *CER10* was six time higher during drought stress as compared to control. Suggesting that these genes have role in wax production during water scarcity stress. High expression of *CER10* and *CER60* wax biosynthesis genes under drought stress indicate that genotype FH-629 is drought tolerance and produce more cuticular wax under drought conditions.

## 4 Discussion

Epicuticular wax plays important role to protect the land plants from biotic and abiotic stresses. Various gene families i.e., *KCS, KCR, FAR, LACS, VLCFA* and *glossy* have been reported in plant species which play diverse role for biosynthesis of VLCFA, wax monomers and wax transportation. Previously various genes engaged in cuticular wax biosynthesis, regulation and transportation have been mapped, cloned and characterized in *A. thaliana* i.e., *CER1, CER2, CER3, CER4, CER5, CER6, CER7, CER10, KCER1, LACS1, MAH1, LTPG1, CFL1, HDG1, WIN1/SHN1, WSD1, DEWAX1, MYB30, MYB40, MYB16, MYB94, MYB96* and *PAS2* (Samuels et al., 2008, Bernard et al., 2012, Lee et al., 2016). Among these genes *CER4, CER6, CER10, MAH1, WSD1* and *FATB* are engaged in cuticular wax biosynthesis whereas, *CER5, CER7, CFL1, HDG1, WIN1/SHN1, WBC11, MYB30, MYB41* and *MYB96* play their role in regulation and transportation (Samuels et al., 2008, Lee and Suh, 2013). Eceriferum (CER) is among the main gene families related to epicuticular wax biosynthesis under stress conditions. A lot of genes belonging to CER super family have been identified and characterized in other plant species such as *A. thaliana*, *G. hersutum, H. vulgare, T. aestivum* and *Malus domestica* (Liu et al., 2021, Haslam et al., 2015, Li et al., 2019, von Wettstein-Knowles, 2020, Wang et al., 2019). However, this family has not been studied in *H. annuus*. Different cuticular wax biosynthesis mutants have been identified in various plant species i.e. *PpCER1-2* in Poa pratensis (Wang et al., 2021); *HvCER1-1, HvCER1-2,* in *Hordeum vulgare* (Richardson et al., 2007); *BnA1.CER4, BnC1.CER4, BnCER1* in *Brassica napus* (Liu et al., 2021, Wang et al., 2019). Previously lot of research has been conducted on wax biosynthesis in *A. thaliana* (Liu et al., 2021, Jenks et al., 2002, Lee and Suh, 2015) *Z. maize* (Lü et al., 2009, Weng et al., 2010) *B. napus* (Liu et al., 2021, Wang et al., 2019) *L. usitissium* (Lee et al., 2014, Tomasi et al., 2017) *T. aestivum* (Doan et al., 2009, Guo et al., 2016) *O. sativa* (Yang et al., 2017) but due to non-sequencing of *H. annuus* genome this economically important crop remained untouched.

Isoelectric point is (pI) the point where overall charge of protein is neutral or zero and this protein property determines the solubility of a protein (Ahmad et al., 2020). After speciation orthologous gene pairs retain their functions (Blanc and Wolfe, 2004). Our results were agreed with (Muhammad et al., 2021, Azeem et al., 2018) who reported that most of the orthologous gene pairs commonly retain their functions after speciation. The results of comparative phylogenetic tree showed that *H. annuus* followed he same trend as other crops do. The term motif is used to describe a part of protein or subsequence that have specific structure and is correlated with a specific biological function (Ahmad et al., 2020). Conserved motifs referred to a part of proteins that is functionally important. Identification of conserved motifs is an important tool to describe the diversification in protein functions (Muhammad Ahmad et al., 2021). Previously conserved motifs for functional diversification have been characterized in *O. sativa, Populus tremula, V. vinifera* and *A. thaliana* (Wang et al., 2015, Lynch and Conery, 2000). The analysis of conserved domains revealed that gene structure and domains were conserved across members of the same phylogenetic group (Muhammad Ahmad et al., 2021). According to our results it was noted that pattern of motifs remained conserved within a clads which are agreed with (Ahmad et al., 2020, Muhammad et al., 2021) who noted similar results in *H. annuus*.

All the *H. annuus* and *A. thaliana* genes were bearing both exons and introns. Variation was noted in intron size for *CER* genes which may be due to chromosomal rearrangements i.e. duplication, inversion and fusions (Li et al., 2016). Two genes *AtCER6-2* and *HanCER6-2* showed no intron. Intron-less genes were previously discovered in *O. sativa*, which might be attributed to an intron loss event during evolution (Xie et al., 2005, Ross et al., 2007). Exon-intron structural diversity is regarded as a useful approach for phylogenetic categorization of CER genes, and is attributed to gene family diversification and evolution (Muhammad et al., 2021, Han et al., 2016).

Chromosomal mapping describes physical location of genes that effect a specific trait (Azeem et al., 2018). Diversity of chromosomal distribution showed that these genes have diverse functions. Recently it has been observed that chromosomal location and gene position is responsible for important characteristics such as carbohydrate accumulation, wax and flavonoid biosynthesis (Masamura et al., 2012, Masuzaki et al., 2006). Previously no study was available for mapping of *CER* genes in *H. annuus* hence results remained un-compared.

Drought stress upregulated the expression of *CER* genes whereas their expression was down regulated during normal supply of water (Wang et al., 2021). Overexpression of *CER1-2* under drought treatment has been reported in *B. napus* (Wang et al., 2019) and P. pratensis (Wang et al., 2021) by enhancing the cuticular permeability and alkane biosynthesis. Recently ten *CER* genes *MdCER1* to *MdCER10* has been characterized for expression in various organs of apple plants. Expression analysis of *MdCER10* in apple has confirmed that this gene showed highest expression in plant leaves (Qi et al., 2019). In cucumber a gene *CsCER* has been evaluated for its role in peal and leaf wax biosynthesis under drought stress (Wang et al., 2015). A series of genes belonging to ECERIFERUM (CER) family i.e., *CER1. CER2, CER3, CER4, CER6 CER10, CER22 and CER60* have been characterized in *A. thaliana* which functions for biosynthesis of wax monomers (Bourdenx et al., 2011, Pascal et al., 2019, Pascal et al., 2013, Bernard et al., 2012). Expression of *CER60* in yeast produces LCFAs having chain length C30 (Trenkamp et al., 2004). In *A. thaliana* overexpression of *AtCER1* has confirmed role in alkane biosynthesis and wax crystallization (Bourdenx et al., 2011, Pascal et al., 2019). Under normal growth conditions wax biosynthesis genes express them at very low level under water stress conditions their expression is upregulated. Co-expression of *CER2* with *CER60* lead to LCFAs synthesis (Haslam et al., 2015).

## 5 Conclusions

Present study was aimed to conduct the genome-wide survey of *H. annuus* CER proteins using *A. thaliana* sequences as query. We detected thirty-seven putative CER sequences in the *H. annuus *genome. We analyzed the phylogenetic relationships, gene structure, chromosomal locations, evolutionary relationship between *A. thaliana* and *H. annuus* genome. Further expression profiling of *CER* genes in *H. annuus* were noted under exposure to drought stress. This is the first study to undertake a genome-wide analysis of CER gene family in *H. annuus*. Two *CER* genes *CER10* and *CER60* showed their expression in all the three cultivars FH-331, FH-629 and FH-630. Results of qRT-PCR showed that these genes were upregulated when the plants were subjected to drought stress. These results will provide valuable information on the functions of CER genes in this crop and will facilitate future studies of evolutionary relationships among *H. annuus* species.

## Funding

The publication of the present work is supported by the Natural Science Basic Research Program of Shaanxi Province (grant no. 2018JQ5218) and the National Natural Science Foundation of China (51809224), Top Young Talents of Shaanxi Special Support Program.

## Declaration of Competing Interest

The authors declare that they have no known competing financial interests or personal relationships that could have appeared to influence the work reported in this paper.
